# Supramaximal high‐intensity interval training improves heart rate variability in older adults: A randomized controlled trial

**DOI:** 10.14814/phy2.70910

**Published:** 2026-06-16

**Authors:** Erik Frykholm, Jesper Boman‐Häggbom, Henrik Holmberg, Bengt Johansson, Sofi Sandström, Emma Simonsson, Urban Wiklund, Erik Rosendahl, Mattias Hedlund

**Affiliations:** ^1^ Department of Community Medicine and Rehabilitation Umeå University Umeå Sweden; ^2^ Department of Epidemiology and Global Health Umeå University Umeå Sweden; ^3^ Department of Diagnostics and Intervention Umeå University Umeå Sweden; ^4^ Umeå Center for Functional Brain Imaging (UFBI) Umeå University Umeå Sweden; ^5^ Department of Diagnostics and Intervention, Biomedical Engineering and Radiation Physics Umeå University Umeå Sweden

**Keywords:** aging, autonomic function, heart rate variability, interval training, parasympathetic activity, vagal tone

## Abstract

Heart rate variability (HRV), a marker of cardiovascular health, declines with aging and physical inactivity. We compared the effects of supramaximal high‐intensity interval training (HIT) with brief intervals and moderate‐intensity training (MIT) on HRV in non‐exercising older adults. Sixty‐eight participants were randomized to 12 weeks of supervised cycling performed twice weekly: supramaximal HIT (10 × 6‐s intervals) or MIT (3 × 8‐min intervals), with training intensity individually prescribed and progressively increased over the intervention using external power output. HRV was assessed using time‐domain indices (SDNN, RMSSD, and LnRMSSD) and resting heart rate from 5‐min segments, derived from 10‐min recordings collected in participants' homes on seven consecutive days at baseline and follow‐up. Between‐group differences in change favored supramaximal HIT for SDNN (5.4 ms [95% CI: 1.1–9.7]), RMSSD (3.5 ms [0.7–6.3]), and LnRMSSD (0.15 [0.01–0.28]). Within‐group changes showed an increase in RMSSD in the HIT group (2.0 ms [0.1–4.0]) and a decrease in SDNN in the MIT group (−3.1 ms [−6.2 to −0.1]). Resting heart rate did not differ between groups. In conclusion, supramaximal HIT induced more favorable HRV adaptations than MIT in non‐exercising older adults.

## Graphical abstract

In older adults, 12 weeks of twice‐weekly supramaximal cycling HIT resulted in more favorable HRV (LnRMSSD) adaptations compared with moderate‐intensity training.

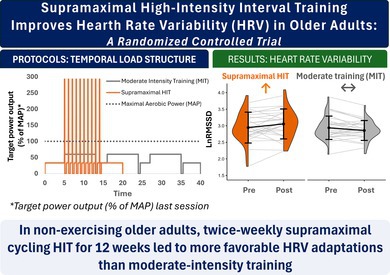

## 1 INTRODUCTION

Cardiovascular disease remains a leading cause of mortality worldwide, accounting for approximately 18 million deaths annually, according to the World Health Organization (2021). The autonomic nervous system regulates a wide range of bodily functions (Karim et al., 2023) and is closely associated with cardiovascular health (Collins et al., 1980). With advancing age, functional changes in autonomic nervous responses have been documented, indicating a gradual decline in autonomic adaptability. This age‐related shift is often exacerbated by physical inactivity, as both factors contribute to alterations in autonomic regulation, typically resulting in autonomic imbalance characterized by increased sympathetic activity and reduced parasympathetic (vagal) tone (Baker et al., 2018; Barnes et al., 2014; Kaye & Esler, 2008; Parashar et al., 2016). This imbalance is recognized as a key marker of elevated cardiovascular risk (Curtis & O'Keefe, 2002; Kikuya et al., 2000; Thayer et al., 2010; Wulsin et al., 2015).

Heart Rate Variability (HRV), defined as the variation in time intervals between consecutive heartbeats, serves as a widely accepted, noninvasive marker of cardiac autonomic function (Camm et al., 1996). While HRV is often interpreted as a general indicator of autonomic balance, the most commonly used indices primarily reflect parasympathetic activity. This makes HRV particularly suitable for detecting reductions in vagal activity that contribute to the autonomic dysregulation associated with aging and sedentary lifestyles, which are typically characterized by increased sympathetic dominance and diminished parasympathetic modulation (Almeida‐Santos et al., 2016; Parashar et al., 2016). In older adults, reduced HRV is associated with cardiovascular morbidity and mortality (de Bruyne et al., 1999; Dekker et al., 1997; Hillebrand et al., 2013; Kikuya et al., 2000), and is considered a valuable indicator of autonomic function, cardiovascular status, and longevity (Curtis & O'Keefe, 2002; Kikuya et al., 2000; Kiviniemi et al., 2014; Thayer et al., 2010; van Biljon et al., 2018; Wulsin et al., 2015). Given its association with autonomic function, HRV is frequently used to evaluate the effects of interventions targeting cardiovascular regulation, such as physical exercise (Buchheit, 2014).

Aerobic exercise training has consistently been shown to shift autonomic balance by reducing sympathetic activity and enhancing vagal tone (Albinet et al., 2010; Earnest et al., 2012; Ståhle et al., 1999). In recent years, supramaximal high‐intensity interval training (HIT) has emerged as a time‐efficient alternative for improving cardiometabolic and physical functional outcomes (Adamson et al., 2014; Babraj et al., 2009; Jakeman et al., 2012; Metcalfe et al., 2012). This form of exercise, most often performed on a cycle ergometer, consists of short, repeated intervals performed at external intensities in watts exceeding the power output associated with peak oxygen uptake (V̇O_2_peak), interspersed with recovery periods (Buchheit & Laursen, 2013).

Hedlund et al. (2019) have introduced a controlled supramaximal HIT protocol adapted for older adults. The protocol consists of short (6‐s), standardized cycling intervals with individualized progression guided by a structured set of criteria, including perceived exertion, performance, and participant readiness. This exercise training model has shown similar to superior effects compared to moderate‐intensity training (MIT) in cardiorespiratory fitness, muscle strength, anaerobic performance, and specific cognitive outcomes, despite requiring less total training time (Frykholm, Hedlund, et al., 2024; Simonsson et al., 2023) in non‐exercising older adults. It has also been reported as applicable, well tolerated, and positively experienced by the older adults (Fridberg et al., 2025; Frykholm, Simonsson, et al., 2024).

Previous HRV studies of interval training in older adults have mainly examined adaptations to long‐term aerobic exercise interventions, including a range of aerobic exercise protocols (Grässler et al., 2021). In younger adults, HRV adaptations to supramaximal interval training have been examined, typically in the context of training interventions with 30 s all‐out bouts (de Sousa et al., 2018; Oliveira et al., 2022) or, in one case, very short (<10 s) efforts (Alansare et al., 2018). To our knowledge, no studies have assessed HRV adaptations to controlled supramaximal interval training in older adults.

This absence of data in older adults makes it relevant to examine HRV responses to controlled supramaximal HIT in this population. Such training consists of repeated brief efforts performed at very high external workloads interspersed with recovery, producing a characteristic cyclic heart rate response within each session (Hedlund et al., 2019). Exercise intensity appears to influence autonomic adaptation (Grässler et al., 2021), and high‐intensity interval training has been associated with greater improvements in vagal‐related HRV than moderate‐intensity continuous training (Alansare et al., 2018; Yang et al., 2024). Accordingly, we hypothesized that controlled supramaximal HIT would induce greater improvements in HRV than MIT. The aim of this study was therefore to compare the effects of a 12‐week program of controlled supramaximal HIT and MIT on HRV in previously non‐exercising older adults.

## 2 MATERIALS AND METHODS

### 2.1 Study design and participants

*The Umeå HIT Study* (ClinicalTrials.gov ID: NCT03765385) was a randomized controlled trial conducted in 2019–2020. Detailed methodology and intervention protocols are described in Simonsson et al. (2023). The present paper presents pre‐specified secondary outcomes focusing on HRV. Changes to the study protocol after its registration included the decision to analyze HRV in the time‐domain indices only, and not in the frequency domain, as well as to only analyze data from baseline and 3‐month follow‐up, omitting the mid‐intervention and 9‐month follow‐up data. The focus on time‐domain indices was motivated by their robustness in unsupervised, home‐based assessments (Shaffer & Ginsberg, 2017) and aligns with recommendations to limit the number of HRV metrics for interpretability and consistency (Laborde et al., 2017). Mid‐intervention testing was omitted to ensure comparable measurement conditions at baseline and follow‐up, and the 9‐month follow‐up data were not analyzed due to missing data as an effect of the Covid‐19 pandemic. Sample size estimation was based on estimated between‐group differences in change for primary outcome variables (V̇O_2_peak) and global cognition. The Regional Ethical Review Board in Umeå, Sweden approved the study (2018‐307‐31M, 2018‐421‐32M), and all participants provided their written informed consent before inclusion. Participants were informed about the general purpose of the study but were blinded to the specific study hypotheses. They received detailed information only about the exercise protocol to which they were assigned. Outcome assessors remained blinded to group allocation throughout the study and all data processing, quality checks, and exclusions were conducted blinded to group allocation, in accordance with the study protocol. Randomization to HIT or MIT was performed after baseline assessments by an independent researcher not involved in study implementation or analysis. Random sequences were generated using *Research Randomizer* (http://www.randomizer.org/) and stratified by biological sex and age group (65–69 or ≥70 years) to ensure balanced allocation.

**FIGURE 1:**
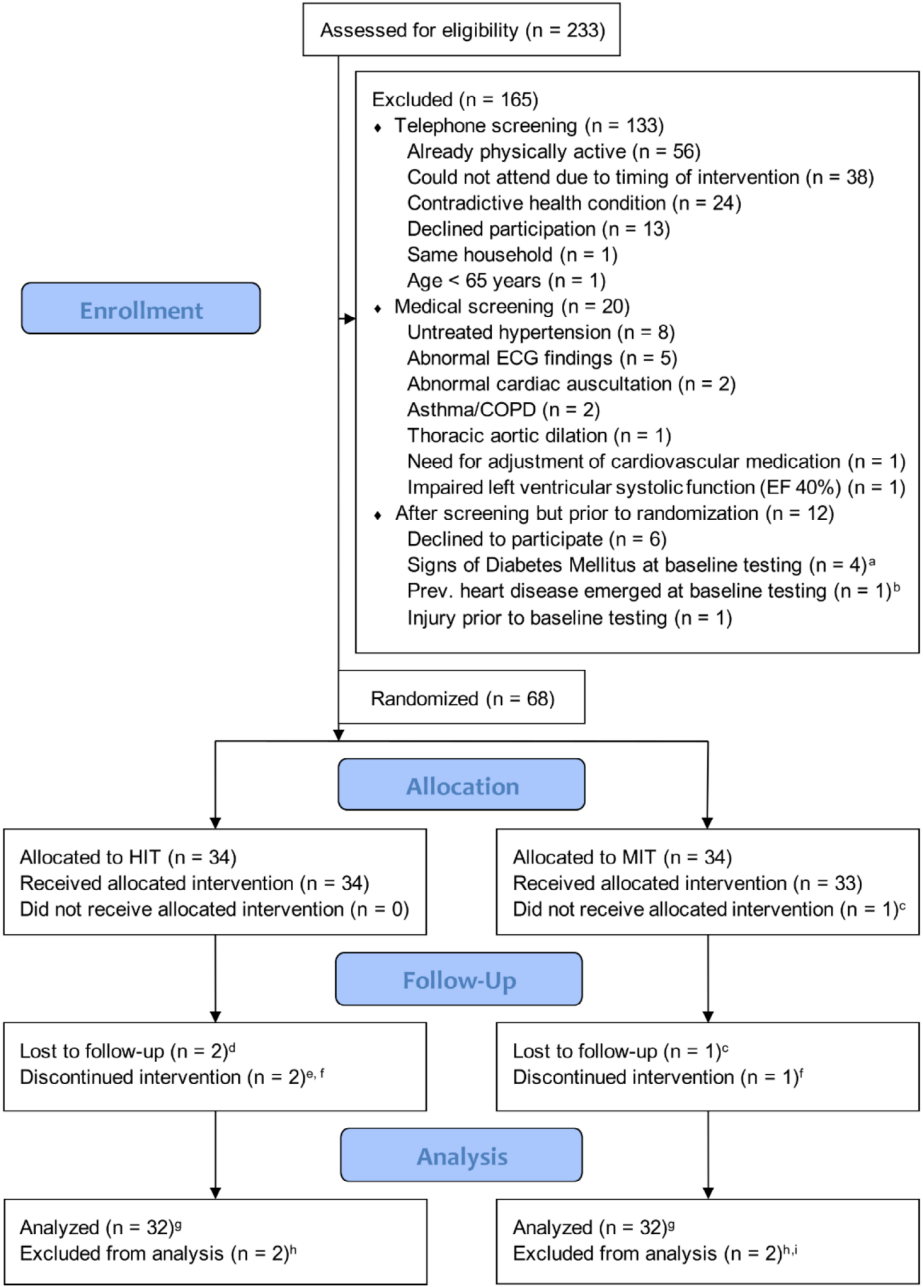
CONSORT flow diagram of Umeå HIT Study COPD, chronic obstructive pulmonary disease; ECG, electrocardiogram; EF, ejection fraction; HIT, controlled supramaximal high‐intensity interval training. (a) Unexpected signs of diabetes mellitus during baseline testing requiring further medical investigation; (b) unexpected history of previous heart disease emerging at the baseline familiarization visit; (c) withdrawal from the study during the intervention without attending any exercise sessions; (d) withdrawal from the study, of which one occurred during the intervention period and one after the intervention but prior to follow‐up; (e) discontinued the intervention at doctor's request (re‐diagnosed with thrombophlebitis) but participated in follow‐up; (f) discontinued the intervention at own request but participated in follow‐up; (g) participants with at least three valid days of HRV recording at baseline, follow‐up, or both; (h) broadband pattern considered to reflect arrhythmia; (i) poor recording quality.

In brief, participants for the Umeå HIT study were recruited through newspaper advertisements targeting older adults in Umeå, Sweden. A total of 233 individuals responded and underwent initial telephone screening. Of these, 100 individuals met the preliminary eligibility criteria and were invited for a comprehensive medical evaluation conducted by an experienced cardiologist. This evaluation included a resting electrocardiogram and clinical assessment (for details, see Simonsson et al. (2023)). Screening was conducted through a telephone interview, medical evaluation, and pre‐baseline testing. Individuals were excluded for reasons such as regular participation in structured exercise training, cognitive impairment (Mini‐Mental State Examination <27), contraindications to high‐intensity exercise (e.g., heart failure, uncontrolled hypertension, myocardial ischemia, significant arrhythmia, or symptoms during cardiopulmonary exercise testing), movement‐related dysfunction, poor blood pressure control or untreated arterial hypertension, insulin‐treated diabetes, chronic or progressive neurological disease, findings during clinical examination or blood sampling warranting further medical investigation, or sharing a household with a participant already enrolled in the study. During the medical evaluation, 20 individuals were excluded. Among those who passed, an additional 12 were excluded during pre‐baseline testing before randomization (Figure 1). In total, 68 participants aged 65 years or older were included in the study.

### 2.2 Baseline and follow‐up testing

Prior to the intervention, participants underwent a cardiopulmonary exercise test (CPET) to determine V̇O_2_peak and corresponding maximal aerobic power (MAP). The test was performed on an electronically braked cycle ergometer (Monark 839E, Monark Exercise AB, Vansbro, Sweden). After an initial workload of 30 W for females and 40 W for males maintained for 3 min, workload increased continuously by 10 W/min for females and 15 W/min for males. V̇O_2_peak was defined as the highest 30‐s average oxygen uptake achieved during the test, and MAP as the corresponding power output. The test was terminated at volitional exhaustion, most commonly corresponding to a rating of perceived exertion ≥18 on the Borg scale, or according to predefined safety criteria; detailed procedures and termination criteria have been described previously (Simonsson et al., 2023).

Additionally, maximal mean power output for 6 s was estimated using a modified Borg Cycle Strength Test, a protocol designed to estimate 6‐s maximal mean power output on a cycle ergometer (De Brandt et al., 2025). These measures were used to prescribe individualized exercise intensities for the intervention.

To describe the participants' physical activity level, the Swedish Board of Health and Welfare's physical activity questionnaire was used, covering both everyday activity and structured exercise. Scores range from 3 to 19, and a value of 11 is equivalent to the WHO recommendation of ≥150 min of moderate‐to‐vigorous activity per week. This tool has been validated against accelerometry and cardiorespiratory fitness (Olsson et al., 2016).

### 2.3 Heart rate variability measurements

HRV was assessed over seven consecutive mornings at participants' homes. Recordings were conducted immediately upon waking, with participants instructed to remain supine, breathe normally, and avoid movement during a 10‐min period. They were also advised to empty their bladder beforehand if needed. Interbeat interval (IBI) data were recorded using a Polar V800 heart rate monitor (1000 Hz) (Polar Electro OY, Kempele, Finland) (Giles et al., 2016) and preprocessed using the Kubios software (Kubios OY, Kuopio, Finland) (Tarvainen et al., 2014). Heartbeats were detected from the pulse wave using a matched filtering approach (Kubios, n.d.). Artifacts (e.g., ectopic or missing beats) were identified by automated algorithms based on IBI deviations (Lipponen & Tarvainen, 2019) and replaced with NaN values without interpolation. Subsequent analyses were conducted in MATLAB (Mathworks Inc., Natick, MA, USA).

Minutes 3–8 of each recording were extracted to ensure stable resting conditions. Segments were considered valid if ≥90% of beats were normal; recordings with excessive arrhythmias were excluded. Validity was confirmed by visual inspection of the IBI data and corresponding power spectrum, where a broadband pattern indicated arrhythmia. HRV was quantified using the time‐domain indices standard deviation of intervals between two normal heartbeats (SDNN), root mean square of successive differences (RMSSD), and the natural logarithm of RMSSD (LnRMSSD), calculated from normal‐to‐normal intervals derived from the IBI data, in line with current recommendations for applied HRV research (Shaffer & Ginsberg, 2017; Yang et al., 2024). Resting heart rate was additionally analyzed as a complementary outcome. Participants with at least three valid recordings at both baseline and follow‐up were included in the analysis. For each time point, HRV metrics were calculated as the mean across valid days. All HRV analyses, including validity checks and exclusion of recordings, were performed by an investigator blinded to group allocation.

### 2.4 Exercise interventions

The exercise protocols have been described in detail previously (Simonsson et al., 2023). In brief, supramaximal HIT and MIT were performed indoors on stationary bikes (Tomahawk IC7) in supervised group sessions (8–10 participants), twice weekly for 3 months (25 sessions). Each session included 5 min of cycling at 30% of maximal aerobic power (MAP) as warm‐up and cooldown.

The supramaximal HIT protocol consisted of 10 × 6‐s intervals interspersed with 54‐s recovery. Initial intensity was set to 60% of estimated 6‐s maximal mean power output (~170% of baseline MAP). The MIT protocol consisted of 3 × 8‐min continuous bouts, initially set to 40% of baseline MAP.

Exercise intensity was prescribed as external target power output in W. Progression followed predefined escalation and de‐escalation criteria (Simonsson et al., 2023) based on perceived exertion, readiness to increase workload, and ability to sustain the prescribed power. Heart rate was continuously monitored (Polar H10), related to maximal heart rate during CPET and incorporated in the progression criteria. Adjustments were made in fixed absolute steps. By the end of the intervention, median interval intensity corresponded to 323% of baseline MAP (interquartile range: 196%–442%) in HIT and 71% (40%–90%) in MIT (Simonsson et al., 2023). Median peak heart rate during training corresponded to 90% of maximal heart rate during baseline CPET (interquartile range: 88%–94%) in session 3 (first session with the full 10‐interval protocol) and 91% (90%–95%) in session 25 in HIT, and to 75% (73%–80%) and 85% (82%–89%) in MIT, respectively.

Session attendance and safety outcomes have been described previously (Simonsson et al., 2023). In brief, attendance was high: 88.0% for supramaximal HIT and 88.1% for MIT. Of 1497 completed sessions, 52 discomfort events were reported in the supramaximal HIT group and 94 in the MIT group, mostly musculoskeletal. No serious adverse events occurred. Details on adherence and progression have been presented (Frykholm, Simonsson, et al., 2024).

### 2.5 Statistical analyses

All statistical analyses were conducted in R (4.5.1. R Foundation for Statistical Computing, Vienna, Austria.), and RStudio (2025.09.1+401, Posit Software, PBC, Boston MA, USA), using the packages *tidyverse* (Wickham et al., 2019), *lme4* (Bates et al., 2015), *emmeans* (Lenth, 2023), and *effectsize* (Ben‐Shachar et al., 2020). All available participant data were analyzed according to the participants' original group allocation in agreement with the intention‐to‐treat principle. Baseline differences between groups were examined using independent samples *t*‐tests for continuous variables and χ^2^ tests or Fisher's exact test for categorical variables. For each outcome, a linear mixed‐effects model with random intercept was applied to examine within‐group changes and between‐group differences in change over time. Group, time (baseline and 3‐month follow‐up), and the group × time interaction were included, with adjustments for age and sex as fixed effects and individual as a random effect. Residuals were visually inspected and judged to meet the underlying assumptions. Results were reported as linear mixed‐effects model estimated mean change and difference in mean change, with 95% confidence interval (95% CI). A 95% CI excluding zero was chosen as an indication of statistical significance. Effect sizes were calculated from model‐based estimates of between‐group differences in change, divided by the unadjusted pooled standard deviation. They were interpreted as small (≥0.2–<0.5), medium (≥0.5–<0.8), and large (≥0.8) (Cohen, 1988).

## 3 RESULTS

Sixty‐four participants contributed to 118 valid measurement averages whereof 54 participants (27 per group) had valid measurement averages at both baseline and follow‐up. Four participants were excluded from both baseline and follow‐up due to frequent arrhythmia (two participants in the supramaximal HIT group and one participant in the MIT group) or insufficient quality of heart rate recordings (one participant in the MIT group). Ten participants were missing at either baseline (two participants in the supramaximal HIT group and one participant in the MIT group) or follow‐up (three participants in the supramaximal HIT group and two participants in the MIT group) or were excluded at baseline (one participant in the MIT group due to insufficient quality of heart rate recordings) or follow‐up (one participant in the MIT group due to frequent arrhythmia) but contributed one valid measurement average (Figure 1). The mean (± standard deviation) of valid recordings was 6.4 ± 0.8. Baseline characteristics of the participants included are presented in Table 1. Among the 64 participants, 37 were female. The mean (± standard deviation) age, body mass index (BMI), and V̇O_2_peak were 69.8 ± 3.0 years, 26.3 ± 4.0 kg/m^2^, and 22.7 ± 4.8 mL/kg/min, respectively at baseline. A total of 34 participants were prescribed antihypertensive medication, of which 8 were taking beta‐blockers.

**TABLE 1:** Baseline characteristics.

	HIT, *n* = 32	MIT, *n* = 32
Age (years)	69.9 ± 3.2	69.6 ± 2.8
Sex (females/males)	18/14	19/13
Height (cm)	171.7 ± 8.4	168.4 ± 8.4
Weight (kg)	77.1 ± 15.2	75.4 ± 14.5
BMI (kg/m^2^)	26.0 ± 3.5	26.5 ± 4.5
BHW activity level (3–19)^a^	9.0 ± 2.6	9.3 ± 3.8
Peak oxygen consumption (L/min)	1.8 ± 0.5	1.6 ± 0.4
Peak oxygen consumption (mL/kg/min)	23.4 ± 5.3	21.9 ± 4.3
Any medication (yes/no)	21/11	23/9
For lowering blood pressure (yes/no)	15/17	19/13
Beta blockers (yes/no)	5/27	3/29
Resting heart rate (bpm)	65.2 ± 8.7	66.0 ± 6.7
SDNN (ms)	43.5 ± 15.6	39.8 ± 9.6
RMSSD (ms)	22.7 ± 12.1	20.2 ± 8.5
LnRMSSD	2.98 ± 0.50	2.91 ± 0.37
Number of recordings (days)	6.6 ± 0.7	6.5 ± 0.8

*Note*: Values are mean ± standard deviation or *n*.

Abbreviations: BMI, body mass index; HIT, controlled supramaximal high‐intensity interval training; LnRMSSD, natural logarithm of root mean square of successive differences; MIT, moderate intensity training; RMSSD, root mean square of successive differences; SDNN, standard deviation of intervals between two normal beats.

^a^ BHW activity level: Assessed using the Swedish National Board of Health and Welfare's validated physical activity questions. The scale ranges from 3 to 19, where a score of 11 corresponds to meeting the international recommendation of ≥150 min of moderate‐to‐vigorous physical activity per week. No statistically significant baseline differences were observed between groups.

**FIGURE 2:**
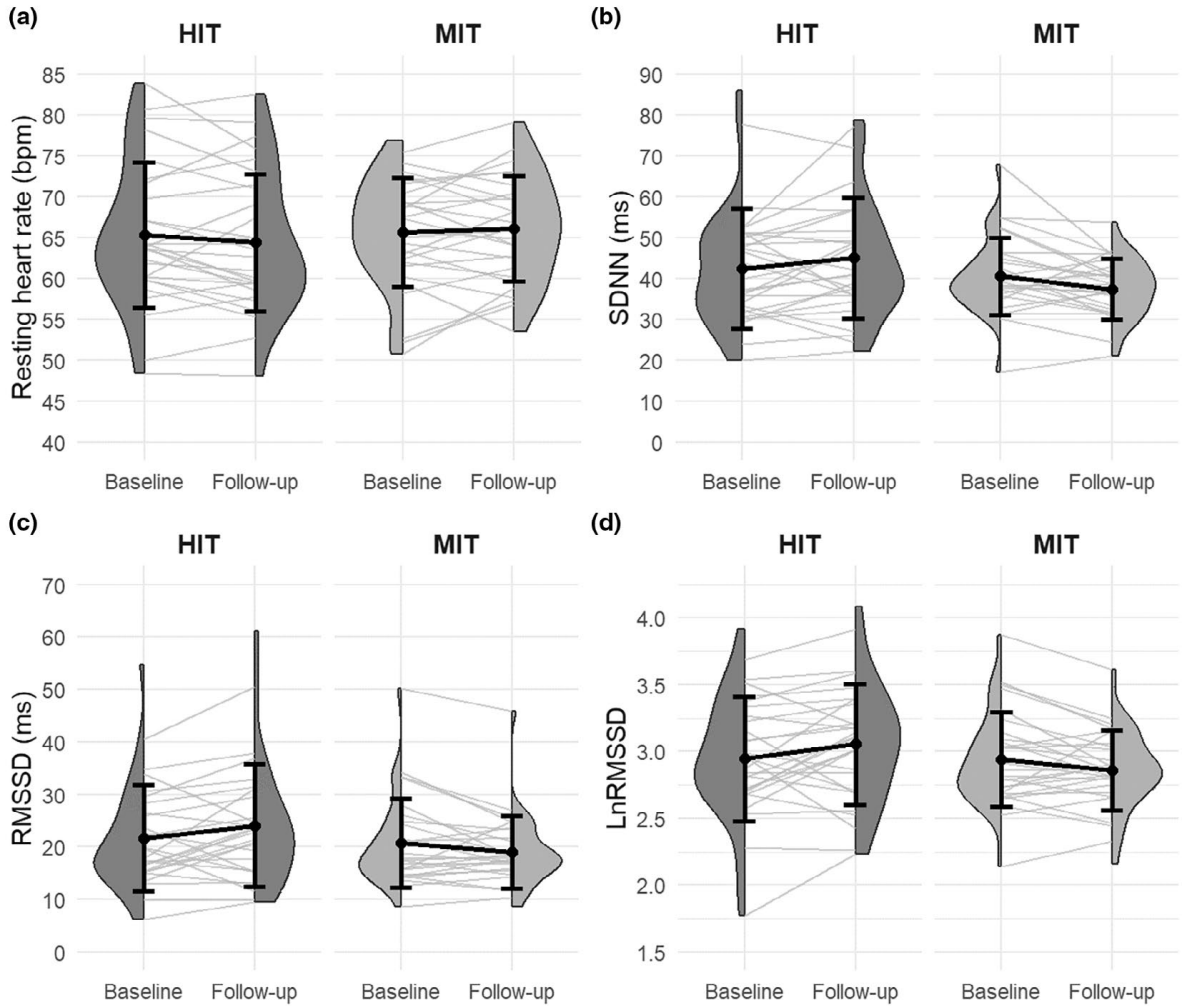
(a) Resting heart rate, (b) SDNN, standard deviation of intervals between two normal heartbeats. (c) RMSSD, root mean square of successive differences, and (d) LnRMSSD, natural logarithm of root mean square of successive differences. Vertical density plots that show a smoothed distribution of data at baseline and follow‐up. For each time point, means were calculated across valid days. Each thin line indicates the change of one individual from baseline to follow‐up. Thick dot and error bars are Group mean ± standard deviation. Thick horizontal line connects mean values.

Figure 2 illustrates the raw data distribution and individual changes in HRV metrics from baseline to follow‐up, along with group means and standard deviations. Estimated marginal means are presented in Table 2. Between‐group differences in HRV change favored the supramaximal HIT group over the MIT group. Specifically, the between‐group differences in change were for SDNN 5.4 ms [95% CI: 1.1, 9.7], RMSSD 3.5 ms [95% CI: 0.7, 6.3], and LnRMSSD 0.15 [95% CI: 0.01, 0.28]. Within‐group changes were an increase in RMSSD in the supramaximal HIT group (2.0 ms [95% CI: 0.1, 4.0]) and a decrease in SDNN in the MIT group (−3.1 ms [95% CI: −6.2, −0.1]). Effect sizes ranged from 0.61 to 0.70. For resting heart rate, the between‐group difference in change was −1.3 beats per minute [95% CI: −3.4, 0.7]. The point estimates moved in opposite directions, with a small decrease in the supramaximal HIT group (−0.8 beats per minute [95% CI: −2.3, 0.7]) and a small increase in the MIT group (0.5 beats per minute [95% CI: −0.9, 2.0]). However, the confidence intervals indicated no difference, either within or between groups.

**TABLE 2:** Within‐group changes and between‐group differences in change from baseline to 3‐month follow‐up.

	Within‐group change				Between‐group difference in change		
	HIT, *n* = 27		MIT, *n* = 27		Group × time		
	Mean	[95% CI]	Mean	[95% CI]	Mean	[95% CI]	ES
Resting heart rate (bpm)	−0.8	[−2.3; 0.7]	0.5	[−0.9; 2.0]	−1.3	[−3.4; 0.7]	−0.35
SDNN^a^ (ms)	2.3	[−0.8; 5.3]	−3.1	[−6.2; −0.1]	5.4	[1.1; 9.7]	0.69
RMSSD^b^ (ms)	2.0	[0.1; 4.0]	−1.5	[−3.5; 0.5]	3.5	[0.7; 6.3]	0.70
LnRMSSD^c^	0.09	[−0.01; 0.18]	−0.06	[−0.16; 0.03]	0.15	[0.01; 0.28]	0.61

*Note*: Estimated marginal means from linear mixed‐effects models with a random intercept with adjustments for age and sex as fixed effects and individual as a random effect. Residuals were visually inspected and judged to meet the underlying assumptions. Reported as change within‐groups, and between‐group difference in change with 95% confidence intervals. Effect size was calculated from the model estimates of between‐group difference in mean change and the unadjusted pooled standard deviation.

Abbreviations: CI, confidence interval; ES, effect size; HIT, high‐intensity interval training; MIT, moderate‐intensity training; *n*, number of participants with available data at both timepoints.

^a^ Standard deviation of intervals between two normal heartbeats.

^b^ Root mean square of successive differences.

^c^ Natural logarithm of root mean square of successive differences.

## 4 DISCUSSION

The primary finding is that in non‐exercising older adults, 12 weeks of controlled supramaximal HIT resulted in improvements in HRV compared to MIT. Between‐group analyses showed consistent differences across all time‐domain HRV metrics (SDNN, RMSSD, and LnRMSSD), with effect sizes in the medium range. Within‐group patterns moved in coherent directions, with increases in RMSSD in the supramaximal HIT group and decreases in SDNN in the MIT group. For resting heart rate, the confidence intervals indicated no difference within or between groups, although the point estimates moved in opposite directions (a small decrease in the supramaximal HIT group and a small increase in the MIT group). Taken together, these findings suggest that, in this population, supramaximal HIT may promote more favorable autonomic adaptations than MIT.

Our novel findings in non‐exercising older adults are supported by previous studies demonstrating that training intensity is a key determinant of autonomic adaptation in other populations, with higher‐intensity exercise more effectively improving HRV (Grässler et al., 2021). A recent network meta‐analysis further identified high‐intensity interval training as the most effective modality for enhancing HRV parameters, including SDNN and RMSSD, compared to aerobic, resistance, and combined training (Yang et al., 2024). Also, in physically inactive adults, short high‐intensity intervals have demonstrated superior effects on HRV compared to moderate‐intensity continuous training (Alansare et al., 2018). For resting heart rate, the confidence intervals indicated no differences within or between groups. The reduction following supramaximal HIT was small and likely of limited clinical relevance in isolation, although the direction of change was consistent with the HRV findings, with a small decrease in the HIT group and a slight increase in MIT. Previous work suggests that increases in vagal‐related HRV are often accompanied by lower resting heart rate, although chronotropic adaptations are typically smaller in magnitude and may develop more gradually (Buchheit et al., 2007). Accordingly, the absence of a clear reduction in heart rate does not contradict the HRV results but may indicate that changes in autonomic modulation precede measurable changes in resting heart rate.

The applied importance of our findings is illustrated by both effect size and magnitude of change. Despite the individual variability, the estimated effect sizes clearly favored supramaximal HIT. From an applied perspective, HRV is widely used to monitor training responses, recovery, and readiness to perform (Bellenger et al., 2016), and the observed changes therefore provide insight into how older adults may respond differently to supramaximal HIT compared with MIT. Although the clinical relevance of HRV changes is not yet firmly established, Buchheit (2014) suggested that the smallest worthwhile change in HRV is ~3%. In our sample, group mean changes in HRV indices ranged from 3% to 9% in supramaximal HIT to −2% to –8% in MIT, suggesting potential clinical relevance given established associations between HRV, cardiovascular health, and mortality risk (de Bruyne et al., 1999; Dekker et al., 1997; Hillebrand et al., 2013; Kikuya et al., 2000). Beyond cardiovascular outcomes, HRV has also been linked to other domains, for example, cognitive function and psychological well‐being (Shaffer & Ginsberg, 2017; Spangler et al., 2021), suggesting broader potential relevance for healthy aging.

The mechanisms underlying HRV adaptations to supramaximal HIT and MIT are not fully understood, but the between‐group differences observed here may reflect differences in training intensity, total duration, and temporal load structure. In the present study, supramaximal HIT elicited higher relative peak heart rates and consisted of repeated short bouts of very high external workload interspersed with recovery (Simonsson et al., 2023). Such efforts likely induce marked autonomic perturbations via central command and afferent reflex pathways, including the muscle mechanoreflex, metaboreflex, and arterial baroreflex, leading to pronounced sympathetic activation (Fisher et al., 2015). At the onset of each bout, central command likely contributes to rapid vagal withdrawal and sympathetic activation, while afferent feedback from contracting muscles further modulates cardiovascular responses. The brief efforts, combined with repeated recovery periods, likely facilitate rapid vagal reactivation (Facioli et al., 2021). The repeated occurrence of such activation–recovery cycles may, over time, enhance autonomic regulation, reflected in increased resting HRV.

In contrast, MIT involved longer periods of sustained exercise at moderate intensity. Although moderate‐intensity training is generally associated with stable or improved HRV, group mean HRV declined in the present MIT cohort. Autonomic adaptations have been proposed to follow a hormetic dose–response pattern (Buchheit, 2014), in which the balance between stimulus intensity and duration is critical. Because exercise load reflects both factors (Hofmann & Tschakert, 2017), the longer sustained exposure in MIT, together with achieved heart rate levels, may in some individuals have approached or exceeded the upper range of effective autonomic stimulus. However, the precise mechanisms remain uncertain. The decrease in SDNN should therefore be interpreted cautiously, and without a non‐exercise control group it cannot be determined whether the observed change reflects a true intervention effect or normal physiological variability. Accordingly, the findings should not be interpreted as evidence of a detrimental effect of MIT, but rather as indicating a different autonomic response pattern compared with supramaximal HIT.

Participant characteristics affect the generalizability and applicability of the findings. The mean V̇O_2_peak (~23 mL/kg/min) placed the group in the low‐ to moderate‐fitness range for their age (Kaminsky et al., 2022). Average BMI (~26 kg/m^2^) aligns with national data for this age group in Sweden (Folkhälsomyndigheten, 2021). Although participants were not completely sedentary, self‐reported physical activity levels were, on average, below the threshold corresponding to 150 min per week (Olsson et al., 2016). Notably, five participants in the supramaximal HIT group and three participants in the MIT group were prescribed beta‐blockers, which may influence HRV by attenuating sympathetic and parasympathetic modulation (Coumel et al., 1991), though the numbers were similar in the two groups. Overall, the cohort represents relatively healthy, non‐exercising older adults. Thus, our results cannot be generalized to older adults with significant comorbidities or established cardiovascular disease. Further studies are warranted to assess broader clinical applicability.

### 4.1 Limitations

Some limitations warrant consideration when interpreting these findings. First, HRV was assessed unsupervised in participants' homes over seven consecutive mornings. Although participants were instructed to remain supine and breathe normally, adherence and breathing patterns were not monitored. Home‐based recordings improve feasibility but may introduce variability compared with strictly controlled laboratory assessments. Averaging multiple measurements likely mitigated such fluctuations and provided more robust estimates (Buchheit, 2014; Laborde et al., 2017; Plews et al., 2014). Second, because the home recordings were not fully standardized, we deviated from the original protocol and restricted analyses to time‐domain measures, which are considered robust and well suited for heart‐rate–monitor–based assessments in field settings (Yang et al., 2024). Third, the absence of a passive non‐exercise control group limits the extent to which changes can be attributed solely to the interventions. The observed changes cannot be definitively separated from normal aging‐related drift or measurement variability. Inclusion of a non‐exercise control group would have strengthened causal interpretation. However, the MIT group served as an active comparator and, in our previous work, has shown meaningful improvements in outcomes such as cardiorespiratory fitness and blood pressure (Frykholm, Hedlund, et al., 2024; Simonsson et al., 2023), providing a more realistic reference than a strictly sedentary control condition. This strengthens the practical relevance of the between‐group comparison and highlights their divergent HRV responses. Finally, HRV was a secondary outcome of the Umeå HIT study and the sample was not powered based on HRV endpoints. Precision is therefore limited, particularly given the substantial interindividual variability in HRV responses (Shaffer & Ginsberg, 2017; Yang et al., 2024). Nevertheless, the sample size is comparable to or larger than that of previous HRV exercise interventions in older adults, as summarized in a recent systematic review (Grässler et al., 2021).

## 5 CONCLUSION

In non‐exercising older adults, 12 weeks of controlled supramaximal HIT resulted in resting HRV changes that differed from those seen with MIT. The consistent between‐group differences across all assessed time‐domain HRV indices suggest a shift toward more favorable vagal‐related HRV patterns following supramaximal HIT. Given the age‐related decline in vagal activity and its associations with cardiovascular and cognitive health, these findings support supramaximal HIT as a potential strategy to support vagal‐related autonomic function in older adults.

## AUTHOR CONTRIBUTIONS

**Erik Frykholm:** Formal analysis; methodology. **Jesper Boman‐Häggbom:** Data curation; investigation; project administration. **Henrik Holmberg:** Methodology; supervision. **Bengt Johansson:** Conceptualization; investigation; methodology. **Sofi Sandström:** Data curation; project administration. **Emma Simonsson:** Data curation; investigation; methodology; project administration. **Urban Wiklund:** Data curation; formal analysis; methodology. **Erik Rosendahl:** Conceptualization; funding acquisition; project administration; supervision. **Mattias Hedlund:** Conceptualization; data curation; formal analysis; funding acquisition; investigation; methodology; project administration.

## FUNDING INFORMATION

Swedish Research Council: grant number 2017‐00912; Forte—Swedish Research Council for Health, Working Life and Welfare: grant number 2020‐00159; Kamprad Family Foundation; Seniorhusen Foundation; the Swedish Dementia Association; the Ragnhild and Einar Lundström's Memorial Foundation; the Strategic Research Area Health Care Science (SFO‐V); the Erik and Anne Marie Detlof's Foundation; the Kempe Foundation; the Umeå University Foundation for Medical Research, and Strategic Research Grants 2021 funded by the Faculty of Medicine at Umeå University. The financial sponsors played no role in the design, execution, analysis and interpretation of data or writing of the study.

## CONFLICT OF INTEREST STATEMENT

The authors declare that they have no perceived or potential conflict of interest, financial or otherwise, that could have influenced the presented work.

## ETHICS STATEMENT

The study was approved by the Regional Ethical Review Board in Umeå, Sweden (2018‐307‐31M, 2018‐421‐32M). All participants provided written informed consent before inclusion. The study was conducted in accordance with the Declaration of Helsinki.

## CODE AVAILABILITY

Custom scripts used for preprocessing and analysis of HRV data are available from the corresponding author upon reasonable request for peer review and research purposes, in accordance with institutional and legal regulations.

## Data Availability

Source data for this study are not publicly available due to legal restrictions for sharing sensitive personal information regarding health. The source data are available to verified researchers upon request and with appropriate ethical and legal clearance by contacting the corresponding author.
